# Proteomic Adaptation of *Streptococcus pneumoniae* to the Human Antimicrobial Peptide LL-37

**DOI:** 10.3390/microorganisms8030413

**Published:** 2020-03-14

**Authors:** Pierre-Alexander Mücke, Sandra Maaß, Thomas P. Kohler, Sven Hammerschmidt, Dörte Becher

**Affiliations:** 1 Department of Microbial Proteomics, Institute of Microbiology, Center for Functional Genomics of Microbes, University of Greifswald, Felix-Hausdorff-Str. 8, 17489 Greifswald, Germany; pierre.muecke@uni-greifswald.de (P.-A.M.); sandra.maass@uni-greifswald.de (S.M.); 2 Department of Molecular Genetics and Infection Biology, Interfaculty Institute for Genetics and Functional Genomics, Center for Functional Genomics of Microbes, University of Greifswald, Felix-Hausdorff-Str. 8, 17489 Greifswald, Germany; kohlert@uni-greifswald.de (T.P.K.); sven.hammerschmidt@uni-greifswald.de (S.H.)

**Keywords:** *Streptococcus pneumoniae*, antimicrobial peptide, LL-37, adaptation, proteomics

## Abstract

Secreted antimicrobial peptides (AMPs) are an important part of the human innate immune system and prevent local and systemic infections by inhibiting bacterial growth in a concentration-dependent manner. In the respiratory tract, the cationic peptide LL-37 is one of the most abundant AMPs and capable of building pore complexes in usually negatively charged bacterial membranes, leading to the destruction of bacteria. However, the adaptation mechanisms of several pathogens to LL-37 are already described and are known to weaken the antimicrobial effect of the AMP, for instance, by repulsion, export or degradation of the peptide. This study examines proteome-wide changes in *Streptococcus pneumoniae* D39, the leading cause of bacterial pneumonia, in response to physiological concentrations of LL-37 by high-resolution mass spectrometry. Our data indicate that pneumococci may use some of the known adaptation mechanisms to reduce the effect of LL-37 on their physiology, too. Additionally, several proteins seem to be involved in resistance to AMPs which have not been related to this process before, such as the teichoic acid flippase TacF (SPD_1128). Understanding colonization- and infection-relevant adaptations of the pneumococcus to AMPs, especially LL-37, could finally uncover new drug targets to weaken the burden of this widespread pathogen.

## 1. Introduction

Infections of the lower respiratory tract, especially pneumonia, remain the third leading cause of death and the deadliest communicable disease [1]. Among the causative agents of these diseases is the Gram-positive bacteria *Streptococcus pneumoniae*, the leading cause of bacterial pneumonia [2]. Besides that, *S. pneumoniae* is responsible for other serious diseases like meningitis and septicemia following bacterial invasion into the bloodstream and milder, but even more frequent infections, of the middle ear (otitis media) or sinuses (sinusitis) [3].

Today, two main approaches are used to fight pneumococcal disease. First, vaccination to prevent initial infection, and second, treatment with classical antibiotics. However, the currently available vaccines against *S. pneumoniae* are solely directed against capsule serotypes of selected strains, and debate about the rapid replacement of vaccine-serotypes with non-vaccine-serotypes is rising [4]. On the other hand, the permanent risk for the development of antibiotic-resistant pneumococcal strains makes it necessary to develop new antimicrobial strategies to combat *S. pneumoniae* infections in the future [5].

Interestingly, humans themselves produce endogenous antibiotics called antimicrobial peptides (AMPs) to inhibit infections [6]. In general, antimicrobial peptides are small (12–50 amino acids) amphipathic molecules that exhibit a net-positive charge under physiological conditions [7,8]. Specificity for the bacterial membranes is mainly due to anionic charged phospholipids, in addition to negative charges of LPS in Gram-negative, and teichoic acids in Gram-positive, bacteria. In contrast to that, mammalian membranes predominantly consist of zwitterionic phospholipids. Likewise, the presence of cholesterol reduces the activity of AMPs towards membranes [7,9,10,11,12]. 

The predominant human AMPs are histatins, defensins and a single, 37-amino acid long (4.5 kDa) cathelicidin named LL-37 [13]. While histatins are a class of AMPs found in saliva and are thus just indirectly involved in lung homeostasis [13], several defensins and cathelicidin LL-37 play an essential role in lung innate immunity [14]. Actually, α-defensins are highly present in neutrophils, β-defensins mainly in airway epithelia cells and LL-37 in neutrophils and airway epithelia cells [6,15]. Indeed, LL-37 seems to be the most prominent AMP in bronchoalveolar fluid, with a concentration of up to 25 μg/mL [16], and several studies revealed that LL-37 is clearly involved in defense of the lung against bacterial infections [14]. The best-known effect of LL-37 on bacteria is the perforation of their membranes via toroidal pore formation [7,17,18,19]. Furthermore, LL-37 interrupts the bacterial cell wall synthesis and potentially interacts with pathogenic intracellular protein targets like chaperones [10,20]. Moreover, the disturbance of peptidoglycan synthesis [7] and neutralization of bacterial toxins other than Gram-negative-derived LPS [10] seem plausible. Finally, LL-37 can act as a chemokine to attract leukocytes to the side of infection. This process initiates a diverse immune response against pathogens and elevates the local LL-37 concentration to an even higher level [21].

Considering these modes of action, the antimicrobial peptide LL-37 represents an important stress factor for bacteria, and therefore molecular effectors evolved that reduce the damaging impact of the AMP on the bacterial physiology [9]. Known adaptation mechanisms include the repulsion of LL-37 by cell-surface adaptations, interception or the targeted binding of LL-37 by the release or reconstruction of surface molecules, the export of LL-37 by ABC-transporters and the degradation or modification of LL-37 [7,22]. The repulsion of LL-37 by cell surface adaptations inhibits the initial interaction between the peptide and the bacterial membrane or cell wall. This is mainly accomplished by modifications to the surface charge. The reduction in negative charges or the introduction of positive charges to bacterial membranes and associated structures like teichoic acids and peptidoglycan decreases the attraction of cationic LL-37 [23,24,25]. The interception or targeted binding of LL-37 is thought to be mediated by the release of surface molecules like capsular polysaccharides, teichoic acids and peptidoglycan or the possible restructuring of the peptidoglycan scaffold [16,26,27]. Potentially, the effects of intracellular LL-37 can be counteracted by specific bacterial efflux pumps exporting these peptides [28,29]. In fact, *S. pneumoniae* expresses more than 300 transporters. For a visual overview see: http://www.membranetransport.org/transportDB2/index.html [30]. Lastly, the degradation of LL-37 by proteases, or the hypothetical charge modification of the AMP, would decrease the interaction with the bacterial cell surface. Indeed, this represents a common strategy, that is potentially involved in LL-37 resistance [31,32].

Although adaptations to LL-37 are already described for some bacteria, it remains to be elucidated how *S. pneumoniae*, the leading cause of bacterial pneumonia, reacts to LL-37 stress on a functional level. In this study, gel-free liquid chromatography-mass spectrometry (LC-MS)-based proteomics was used to analyze the proteome of pneumococci under LL-37 stress compared to untreated pneumococci for the first time.

As carriage of *S. pneumoniae* is a prerequisite for infection [3], understanding the interaction between innate immune molecules, specifically AMPs, and pneumococci would give us more opportunities to fight this widespread pathogen. In fact, enhancing the effect of existing antibiotics via the application of antimicrobial peptides was shown and could be an interim solution to fight drug-resistant strains. Presumably, the membrane disruption effect of AMPs promotes access for classical antibiotics into bacterial cells, and this would also explain the synergy between AMPs and antibiotics [7,13,33]. Moreover, resistance against classical antibiotics sensitizes bacteria for antimicrobial peptides [34]. On the other hand, attempts to block the bacterial resistance system to already existing AMPs in the human body continue to be a promising medical approach [23].

## 2. Materials and Methods

**Figure 1:**
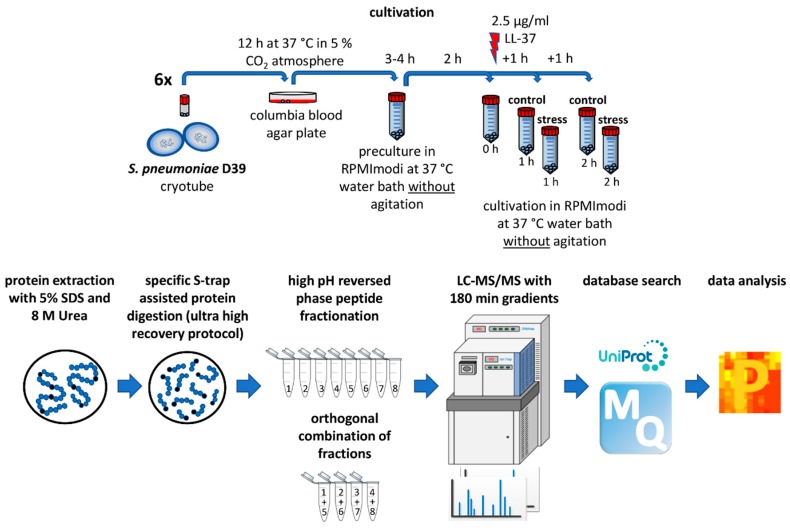
Established workflow for proteomic investigation of LL-37 stress on pneumococci.

For the present study, the following workflow was established by the optimization of numerous parameters including cultivation, protein extraction, digestion, peptide fractionation, LC-MS/MS methods and database search (Figure 1).

### 2.1. Bacteria and Cultivation

The extensively characterized *Streptococcus pneumoniae* D39 strain (serotype 2, NCTC 7466) was taken from cryo culture and incubated for 12 h on Columbia blood agar plates at 37 °C under 5% CO_2_ atmosphere. Bacteria were then transferred for pre-cultivation to 40 mL modified RPMI 1640 media (Thermo Fisher Scientific, Rockford, IL, USA) (further named chemically defined media = CDM) [35] and the bacterial growth was photometrically observed while cultivating at 37 °C in a water bath without agitation. When the culture reached an optical density (OD_600_) of 0.5, cells were diluted in 40 mL of CDM to a starting OD_600_ of 0.07 to receive the main culture. Cultivation was performed under the same parameters as before. After 2 h of bacterial growth, 2.5 μg/mL LL-37 acetate (Innovagen, Lund, Sweden) was added to the stress samples. For controls, an equivalent volume of distilled water (100 μL) was added. Finally, pneumococci were harvested by centrifugation (25 min, 10,000× *g*, 4 °C, soft deceleration) directly before the addition of stress and after 1 and 2 h of LL-37 treatment together with the corresponding control samples. Bacterial pellets were frozen at −80 °C until further processing. Experiments were performed in six independent biological replicates.

### 2.2. Colony Forming Unit (CFU) Assay

Bacteria were grown, stressed and harvested as described before (Section 2.1) in three biological replicates. Following that, cell pellets were diluted to an OD_600_ of 1 in 1 mL sterile phosphate-buffered saline (PBS) [36] corresponding to about 10^9^ bacteria. A total of 100 μL of further (1:100,000) diluted samples were pipetted on blood agar plates. The plates were homogenously covered with bacterial suspension using glass pearls. After the removal of pearls, the plates were incubated for 11 h at 37 °C under 5% CO_2_ atmosphere and the colonies were counted.

### 2.3. LL-37 Sensitivity Testing

*S. pneumoniae* D39 wildtype (wt), as well as Δ*dltD* and Δ*licD2* deletion mutants, were cultivated and stressed as mentioned before (Section 2.1). For evaluating the sensitivity of pneumococci towards LL-37, levels of reduction in optical density caused by the peptide were measured in triplicate experiments in the three strains studied. Differences between mutant and wt inhibition were statistically analyzed using one-tailed *t*-tests assuming unequal variance, *p* < 0.05.

### 2.4. Mutant Construction

The pneumococcal *licD2* mutant in *S. pneumoniae* D39 was constructed by amplification of a DNA fragment consisting of the *S. pneumoniae* D39 l*icD2* gene and ~500 bp up and downstream flanking regions using polymerase chain reaction (PCR) with primer LicD2*Sph*Ifor (5´-GCGCGCGCAT GCTATTGATA CTCGAAATAC AAAAACCTAT G-3´) and LicD2*Sac*Irev (5´-GCGCGCGAGC TCTGGTAAGA TTGGTGATGA CGATAAGG-3´). The purified PCR product was cloned into plasmid pUC18 (Thermo Fisher Scientific, Rockford, IL, USA). The resulting plasmid was used as a template for an inverse PCR using primer Invrev1130*Bam*HI (5´-GCGCGCGGAT CCCCTAATCC TCCAATTTAT AAGCG-3´) and Invfor1130*Sma*I (5´-GCGCGCCCCG GGTTGAGGGG GATTATACAA ACTAC-3´). Afterwards, an *ermB* gene, amplified by PCR from vector pTP1 [37] using primer Invrev*Bam*HIErm (5´-GCGCGCGGAT CCACGGTTCG TGTTCGTGCT GACTTGC-3´) and Infor*Sma*IErm (5´-GCGCGCCCCG GGGTAGGCGC TAGGGACCTC TTTAGC-3´) was inserted. The final recombinant plasmid was used to transform and mutagenize *S. pneumoniae* D39.

For the construction of *S. pneumoniae* D39∆*dltD*, a DNA fragment containing the *dltD* gene and ~500 bp up and downstream of *dltD* was amplified by PCR using Primer DltDfor*XbaI* (5´-GCTCTAGACG AGTGGTCCAA TCGATCGCTT TAAGC-3´) and Dltdrev*Sac*I (5´-GCGAGCTCCC AAGCGTTTGT CTCGATGTTC CCACATG-3´). The purified PCR fragment was cloned into the pMiniT vector (NEB) and the resulting vector was used as a template for an inverse PCR with primer Inv*Kpn*I (5´-GCGCTCTAGA GCGTTTAAGC ATTTTGTAGC TC-3´) and Inv*Nco*I (5´-GCGCCCATGG GATGGAGATG TCAAAGAATT TCAAT-3´). The *ermB* gene, amplified from plasmid pTP1 [37] using primer ermforw (5´-GATGATGATG ATCCCGGGTA CCAAGCTTGA ATTCACGGTT CGTGTTCGTG CTG-3´) and ermrev*Nco*I (5´-GCGCCCATGG CGTAGGCGCT AGGGACCTC-3´) was cloned into the inverse PCR product. The final vector was used to transform and mutagenize *S. pneumoniae* D39.

### 2.5. Sample Preparation for LC-MS Measurements

#### 2.5.1. Protein Extraction and Determination

Loss of hydrophobic proteins during protein extraction was minimized by resuspension of pneumococcal pellets in 1 mL 50 mM triethylammonium bicarbonate (TEAB) lysis buffer (pH 7.55) containing 5% SDS (wt/wt) and 8 M urea. Subsequently, the bacterial suspension was pipetted to cyro tubes filled with 500 μL glass beads (diameter: 0.10 - 0.11 mm). Cells were disrupted by bead beating (6 m/s, 4 × 30 s with 120 s breaks on ice) and subsequent sonication (1 × 60 s). In order to separate the protein-containing supernatant from cell debris, a centrifugation step was performed (8 min, 13,000× *g*, 4 °C).

Protein content was determined using BCA-assays (Pierce BCA Protein Assay Kit; Thermo Fisher Scientific, Rockford, IL, USA) following the manufacturers protocol.

#### 2.5.2. Protein Digestion

For tryptic digestion of 50 μg protein, S-trap spin columns (Protifi, Huntington, NY, USA) were used in combination with the manufactures “ultra-high recovery protocol” available under https://www.protifi.com/resources. Shortly, protein samples were reduced and alkylated and loaded into the suspension traps. Then, the protein solution was acidified, and trypsin added (Promega, Madison, WI, USA) and immediately washed several times. After the addition of further trypsin to the matrix, the trap was incubated for 1 h at 47 °C, enabling protein digestion. Lastly, peptides were eluted with acetonitrile and dried using a vacuum concentrator. 

#### 2.5.3. Peptide Fractionation

To increase the proteomic coverage, peptides were fractionated following the basic pH reversed-phase peptide fractionation protocol (Thermo Fisher Scientific, Rockford, IL, USA) using self-packed columns filled with 18 μg of Reprosil-Gold 300 C18, 5 µm material (Dr. Maisch, Ammerbuch-Entringen, Germany). Basically, peptides were loaded into the column, washed and eluted into eight different fractions using increasing concentrations of acetonitrile in a high-pH solution (0.1% triethylamine). Afterwards, the fractions 1&5, 2&6, 3&7 and 4&8 were orthogonally combined for optimal use of LC-MS analysis time according to the principles described elsewhere [38].

Finally, peptides were dried and suspended in 40 μL 0.5× iRTs in water with 0.1% acetic acid (Biognosys, Schlieren, Switzerland) for LC-MS quality control purposes.

### 2.6. LC-MS Measurements

Samples were measured on an LTQ Orbitrap Velos mass spectrometer in combination with an EASY nLC-1000 liquid chromatography system. First, 2 μL of the sample was loaded with 12 μL buffer A (water with 0.1% acetic acid) on an in-house packed C18 column (ReproSil-Pur 120 C18-AQ, 3 μm material) of 20 cm length and 100 μm inner diameter. Then, a non-linear 180-min gradient from 1% to 99% buffer B (acetonitrile with 0.1% acetic acid) was used for the elution of peptides with a flowrate of 300 nl/min. The MS measurement was performed in data-dependent mode. The MS1 scans covered a mass range of 300–1700 *m*/*z* at a resolution of 30,000 (at 400 *m*/*z*) and the 20 most intense precursors were selected and fragmented via collision-induced dissociation (CID) using a normalized collision energy of 35. Ions with unknown charge or charge one were excluded from fragmentation. Fragment ions were measured in the ion trap (MS2). Additionally, precursors were excluded for 20 s after fragmentation for additional fragmentation.

### 2.7. Quality Control and Data Analysis

#### 2.7.1. Quality Control

Data quality was monitored by adding synthetic iRT peptides (Biognosys, Schlieren, Switzerland) to the samples, thereby checking for potential shifts in mass deviation or retention time. Additionally, MaxQuant output files and RawMeat (VAST Scientific, Cambridge, MA, USA) were used to detect unexpected changes in intensity, the number of obtained scans, the distribution of TopN or identification rates. Finally, hierarchical clustering and a principle component analysis confirmed the quality of our data, showing clustering of replicates, sample points and conditions (Figure S1). Only data passing the quality checks were used in this study.

#### 2.7.2. Protein Identification and Quantification

MaxQuant version 1.6.5.0 and its Andromeda search engine [39] was used for protein identification and quantification with the following settings. Trypsin/P was selected as a protease that cleaves after lysine and arginine independent of following prolines. The maximal number of missed cleavages was set to 2. Likewise, the minimal number of unique peptides per protein group was set to 2 to be considered as identified. Oxidation (M) was considered as a variable and carbamidomethyl (C) as a fixed modification, whereas the maximal number of modifications per peptide was 5. For quantification, solely unique peptides were used. Databases loaded to MaxQuant were: the *S. pneumoniae* D39 proteome containing 1915 proteins obtained from Uniprot.org (Proteome ID: UP000001452, last modified November 9, 2018), the MaxQuant contaminants file and sequences for human LL-37, yeast enolase and iRT peptides.

#### 2.7.3. Data Evaluation

Data were analyzed using Perseus [40] version 1.6.5.0. Initially, the quantitative values were transformed using the function: log_2_(x). Next, values were filtered based on the criteria: only identified by site, reverse, potential contamination, identified in at least 5/6 biological replicates under at least 1/5 condition. Missing values were imputed from normal distribution using the Perseus standard settings. For examination of differences between stress and control samples, Student’s *t*-tests were used (S0: 0, side: both, use for truncation: *p*-value, threshold *p*-value: 0.01). Furthermore, a minimum fold change of 1.5 was defined to extract the most prominent proteomic differences between the tested conditions, which are expected to have an impact on physiology. Lastly, protein annotations were made using the *S. pneumoniae* D39 proteome obtained from Uniprot.org (Proteome ID: UP000001452, last modified November 9, 2018) supplemented with data obtained from PneumoBrowse [41], the subcellular localization database PSORTdb 3.0 [42] and information on the two-component regulatory system CiaHR [43].

### 2.8. Data Availability

The mass spectrometry proteomics data have been deposited to the ProteomeXchange Consortium (http://proteomecentral.proteomexchange.org) via the PRIDE [44] partner repository with the dataset identifier PXD016511.

## 3. Results

To investigate the proteomic response of pneumococci towards LL-37, *S. pneumoniae* D39 was stressed by the addition of 2.5 μg/mL LL-37 during the exponential growth phase (two hours after the inoculation of main cultures) that was expected to be physiologically relevant. Furthermore, pre-experiments showed a clear and concentration-dependent antibacterial effect of the peptide, ranging from a profound inhibition of bacterial growth using 20 μg/mL of LL-37 to almost no inhibition by adding 0.625 μg/mL peptide to pneumococcal cultures.

**Figure 2:**
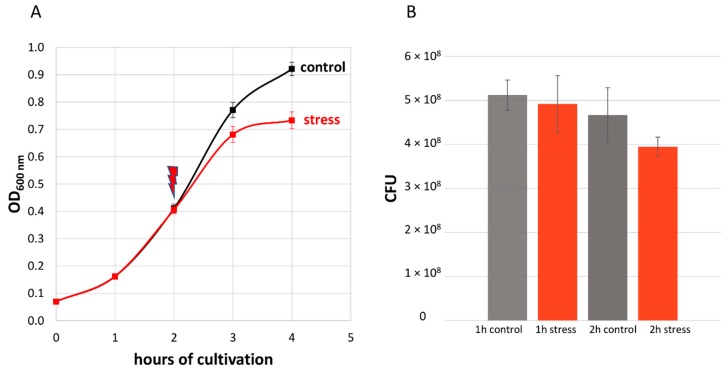
(**A**) Effect of 2.5 μg/mL LL-37 on the growth of *S. pneumoniae* D39. The red arrow indicates the application of the stress after two hours of cultivation. Error bars represent the standard deviation. *n* = 6. (**B**) Colony-forming units (CFU) per ml OD_600_ = 1 bacterial suspension. Samples were taken every hour after the addition of 2.5 μg/mL LL-37. Means are shown and error bars represent the standard deviation. *n* = 3.

In our experiment, the application of stress reduced the maximal optical density (OD_600_) of the cultures by about 20% (Figure 2A). Moreover, the viability of stressed cells was confirmed via colony-forming unit (CFU) assays (Figure 2B). Five samples, 1× before the addition of LL-37, 2× after 1 h of stress (control, stress) and 2× after 2 h of stress (control, stress), were harvested in six biological replicates and prepared for MS, as shown in Figure 1. This allowed us to link the significant reduction in optical density of the stressed bacterial cultures to their proteomic state.

**Figure 3:**
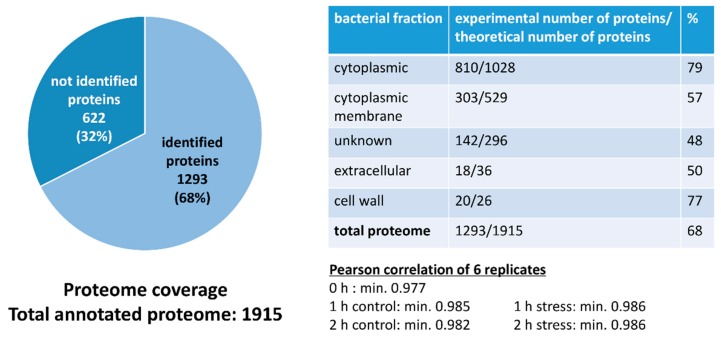
Graphical representation of proteome coverage and correlation of replicates.

In total, 68% of the pneumococcal proteome (1293/1915 proteins) could be identified. Proteins annotated to be cytoplasmic or cell-wall-bound could be identified as almost 80%. Additionally, membrane proteins, extracellular proteins and proteins of unknown localization were covered to about 50%. Finally, the quantitative correlation between the data of biological replicates was at least 97.7% (Figure 3, Figures S2–S6), demonstrating the high reproducibility of our experimental approach.

**Figure 4:**
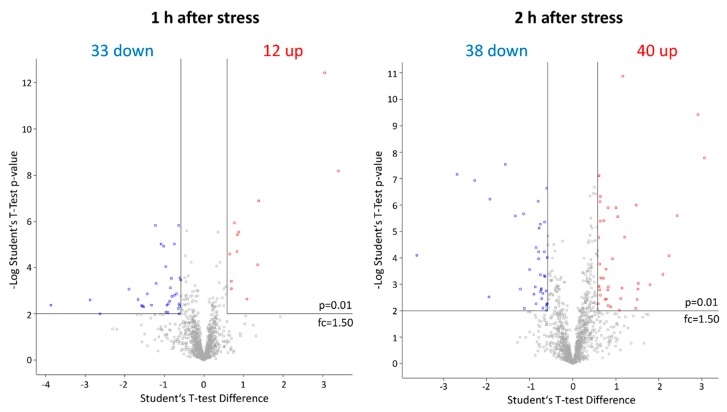
Graphical representation of significantly# affected proteins after LL-37 treatment via Volcano plots. Proteins with an increase in abundance after application of the stress are labeled in red and proteins with a decrease in protein abundance in blue, respectively. # Student’s *t*-test (*p*-value= 0.01, min. fold change = 1.5).

As the obtained dataset passed all executed quality control steps, Student’s *t*-tests were used for the evaluation of differences in the proteome of stressed and unstressed pneumococci consisting of 1118 proteins after value-filtering in Perseus. One hour after application of AMP stress, 45 proteins were significantly changed in abundance (33 less abundant, 12 higher abundant) and 2 h after stress 78 proteins (38 less abundant, 40 higher abundant) fulfilled the strict *t*-test’s criteria to be considered as changed (Figure 4). Furthermore, many proteins that were changed after 1h of stress were also found to be significantly changed in abundance after 2h hours of stress in a similar manner.

**Figure 5:**
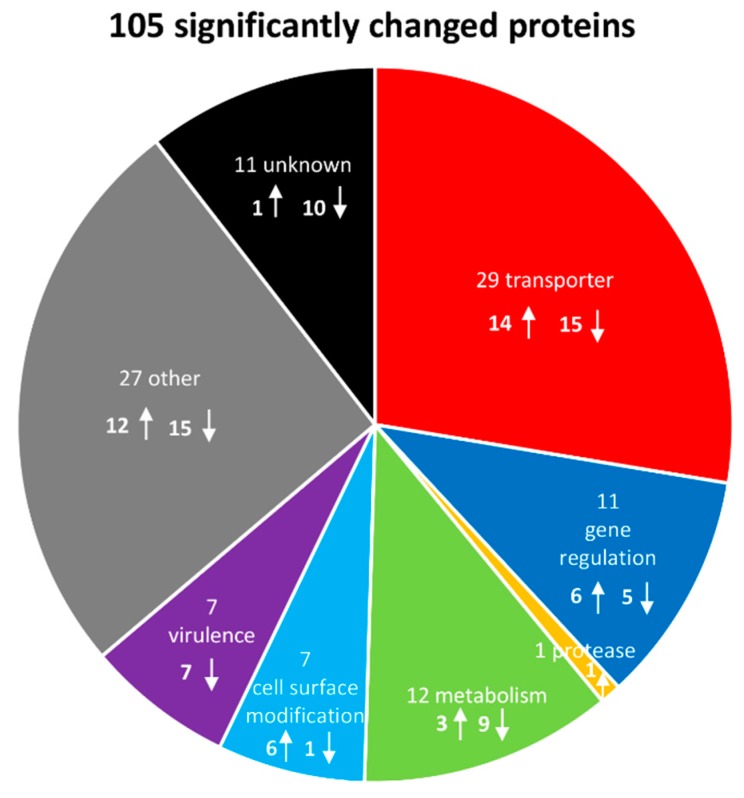
Graphical representation of significantly# affected proteins and their function after LL-37 treatment. The arrows indicate an increase or a decrease in protein abundance after application of the stress, respectively. # Student’s *t*-test (*p*-value = 0.01, min. fold change = 1.5).

Subsequently, significantly changed proteins could be classified into functional groups: transporters (29 proteins), proteins involved in gene regulation (11), a single protease (1), metabolic proteins (12), cell surface modification proteins (7), virulence factors (7) and uncategorized proteins (other (27) and unknown (11)). Hence, a total number of 105 proteins with a significantly changed amount (43 lower in abundance, 62 higher abundant) could be identified after the addition of LL-37 (Figure 5, Table S1).

**Figure 6:**
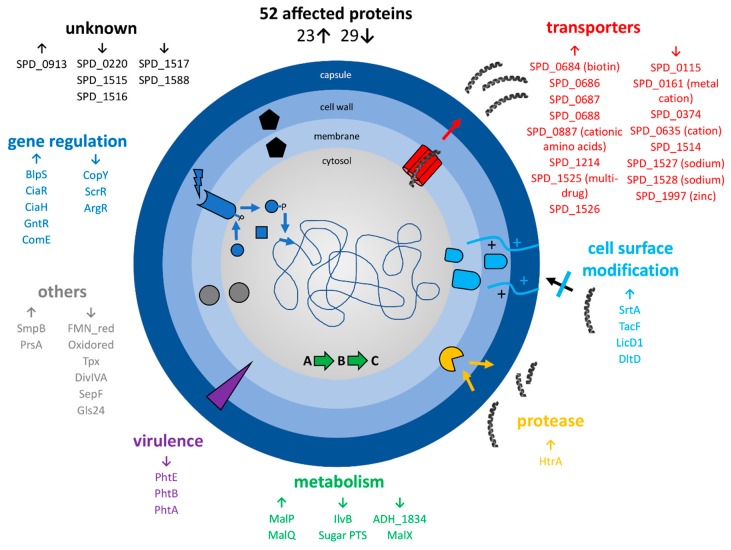
Summary of selected proteomic changes of *S. pneumoniae* D39 upon LL-37 exposure after comparison of significant# proteome changes with published transcriptome and genome data. The arrows indicate an increase or a decrease in protein abundance after application of the stress, respectively. # Student’s *t*-test (*p*-value= 0.01, min. fold change= 1.5).

Before in-depth analyses, the proteome data were compared to publicly available high-quality genomics and gene regulation data for the pneumococcus [41,43] and a transcriptomics dataset for pneumococci after LL-37 stress [45]. Briefly, proteins were considered for further analyzation if at least two criteria were fulfilled. These include bacterial resistance candidates with significantly changed abundances on proteome and transcriptome levels, proteins that were found to be changed at both timepoints on proteome level (after 1h and 2h of stress), hits of several proteins of a single operon, or if levels of a protein and its corresponding regulator were significantly altered after LL-37 stress application. This resulted in a new, extensively examined dataset (Figure 6, Table S2).

Basically, the abundance of many transporters, especially ABC-transporters, changed significantly and often drastically after LL-37 exposure. Eight proteins annotated as transporters were increased in abundance and eight proteins of this class decreased. Most prominent was the more than 8-fold increase in the multidrug ABC transporter SPD_1525-26 after 2h of stress (7.5-fold after 1h of stress) and the significant decrease in SPD_1527-28 sodium ABC exporter (down to 0.4-fold after 1h of stress and 0.3-fold after 2h of stress for SPD_1527). Likewise, the transcriptional regulator SPD_1524 (GntR) was found in considerably higher abundance after LL-37 stress. Additionally, the sensor histidine kinase CiaH and the response regulator CiaR increased after stress at both timepoints. Other proteins involved in gene regulation with a moderately higher level after LL-37 treatment are BlpS (fold change (fc) of 1.8 at the second timepoint) and ComE (fc of 1.5 at the same sample point). Besides that, the heat-inducible serine protease and chaperone HtrA was highly enriched in stress samples (fc of 2.6 and 2.0, 1h and 2h after stress, respectively) and the bacterial metabolism was clearly affected by LL-37, illustrated especially by higher amounts of MalP, MalQ and lower amounts of MalX. While proteins involved in cell surface modification (DltD, LicD1, TacF, SrtA) showed significantly higher abundance under stress, particularly after 2 h of stress, several virulence factors with a role in adhesion (histidine triad proteins A, B and E) were less abundant upon stress with LL-37. Furthermore, proteins involved in correct protein-folding (foldase PrsA) and biosynthesis (SsrA-binding protein SmpB) were more abundant, and proteins involved in cell redox homeostasis (Tpx) and cell division (SepF, DivIVA) were less abundant after LL-37 exposure. Lastly, the unknown proteins SPD_0913 (accumulated up to a fc of 2.8) and SPD_1515-17 (decreased clearly after 2 h of stress) changed significantly.

**Figure 7:**
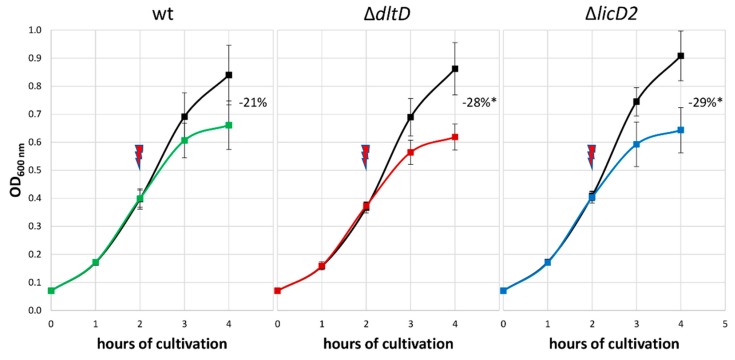
Effect of 2.5 μg/mL LL-37 on the growth of *S. pneumoniae* D39 wildtype (wt) in comparison to stressed D39Δ*dltD* and D39Δ*licD2* mutants. The red arrow indicates the application of the stress after two hours of cultivation. The numbers represent the growth inhibition of stressed pneumococci compared to corresponding untreated D39 strains in percentage. * Significant difference between mutant and wt inhibition using one-tailed t-tests assuming unequal variance, *p* < 0.05. The error bars represent the standard deviation. *n* = 3.

Based on the significant changes seen in the proteomic data, two mutants were chosen and subjected to LL-37 sensitivity testing to support the putative role of the corresponding proteins in LL-37 adaptation (Figure 7). This was done by stressing pneumococci and comparing the reduced optical density of the stressed cells with the ones from the control condition. First, the sensitivity of the *S. pneumoniae* D39Δ*dltD* mutant towards LL-37 was evaluated, as DltD is known to effect resistance to AMPs in pneumococci [46] and was also found to be more abundant in our dataset after LL-37 treatment. Additionally, *S. pneumoniae* D39Δ*licD2* was selected because LicD2 showed a similar change in abundance after LL-37 stress to the significantly increased genomic neighbor LicD1 (without passing the strict statistical parameters). Moreover, a deficiency in *licD1* appears to be lethal [47]. As shown in Figure 7, the growth of the wildtype D39 strain is reduced by about 21% after peptide stress compared to the control condition, whereas growth of Δ*dltD* and Δ*licD2* mutants are significantly reduced by about 28% and 29% in comparison to the untreated strains, respectively.

## 4. Discussion

The presented dataset shows the highest proteome coverage for the *S. pneumoniae* D39 wildtype known to us. Furthermore, 105 proteins could be identified as significantly changed after LL-37 stress, while stressed bacteria showed a similar but about 20% reduced growth compared to unstressed cells. A total of 52 protein candidates with a potential specific function in LL-37 adaptation could be identified after comparison with transcriptomic and genomic data.

Among these candidates are the teichoic acid flippase TacF (SPD_1128) [48] and LicD1 (SPD_1129), an enzyme responsible for transfer of phosphorylcholine to teichoic acids, thereby mimicking eukaryotic membranes, increasing the surface charge and adding binding sites for choline binding proteins to the cell wall [24,49]. Interestingly, both corresponding RNA levels [45] and the protein abundance after 1 h of stress are not significantly affected, but TacF and LicD1 proteins are significantly more abundant after 2 h of stress, suggesting a relatively slow regulation of protein level. Additionally, DltD (SPD_2002), which alanylates teichoic acids and hence introduces positive charges to the cell surface [46], was more abundant in stressed bacteria (after 1 and 2 h) in accordance with the accumulation of its RNA in the transcriptomic dataset [45]. Next, the enzyme sortase SrtA is more abundant under 1 and 2 h of stress, attaching LPXTG-motif proteins to the cell surface. Taking all that into account, it seems plausible that pneumococci are also able to repel the positively charged LL-37 by cell wall adaptations. In fact, a reduction in negative surface charge would decrease the electrostatical forces between the cationic peptide (charge +6) and the bacteria, and thus decrease its antimicrobial effects. This appears even more likely when considering that involvement of the *dlt*- and *lic*-operons in resistance to LL-37 in other bacteria or of *S. pneumoniae* against other cationic antimicrobial peptides was already described. In fact, non-functional *dlt* affected the resistance of *S. pneumoniae* to the bacterial secreted lantibiotics nisin and gallidermin [46], and a *lic* mutation the susceptibility of *Haemophilus influenzae* towards LL-37 [24]. Alternatively, LTA release, aiming for targeted binding of LL-37, would be an effective interception mechanism against the action of the peptide [27], but was not examined in this study.

Furthermore, the abundance of several transporters, especially of the ABC type, was changed in response to LL-37. Consistently using the annotation from TransportDB 2.0, the proteins with transporter function appear to be enriched among the significantly changed proteins (23 out of 105; 22%), as there are 304 transporters annotated for the pneumococcal proteome (16%). The same trend can be observed comparing the number of significantly changed transporters with the transporters identified in our experiment (194 among 1293; 15%). These changes could lead to the removal of LL-37 from the bacterial membrane, as shown for other bacteria and other cationic antimicrobial peptides [50,51,52]. Interestingly, the accumulated transporter proteins are mainly regulated by GntR (SPD_0686-88, SPD_1525-26) and the depleted ones by ComE (SPD_0374, SPD_1527-28), regulators that are both more abundant after the addition of LL-37. The observation that many of the transporters increased after stress, including the ones independent of GntR, has also been made on the RNA level [45]. 

Besides that, the serine protease and chaperone HtrA (SPD_2068) is highly enriched in stress samples. This finding could be explained by a potential LL-37-degrading function of the enzyme, an enhanced removal of damaged or misfolded proteins in response to cell wall stress, or the role of HtrA in competence (ComC degradation) and bacteriocin (BlpC degradation) regulation [53].

Then, the amount of proteins encoded by the maltose operon (MalP, MalQ) probably changed due to changes in CiaR amounts. However, how this observation is connected to AMP adaptation is still unclear.

Interestingly, three surface virulence proteins, the histidine triad protein A, B and E, are depleted during stress, supposedly to reduce interaction with the immune system [54]. As the virulence proteins are regulated by the Zn^2+^-dependent regulator AdcR, it was unexpected that AdcR did not change significantly. However, posttranslational modifications or ligand-binding could alter the regulator’s activity. Probably for the same reason, LytA was not found to be enriched even when the involvement of this enzyme in LL-37 resistance was described for pneumococci [16]. In fact, LytA activation is regulated by phosphorylation [55], and this could also be the case in this study.

Other proteins found to be regulated are foldase PrsA (accumulated at both sample points but expression was not significantly affected) and several proteins involved in cell redox homeostasis, e.g., the thioredoxin peroxidase Tpx (SPD_1464) (shows the same trend on RNA level [45]), and cell division (SepF, DivIVA) (all three proteins are depleted). As foldases are chaperones and support correct protein folding, presumably there is an enhanced accumulation of misfolded proteins after LL-37 stress in pneumococci. Furthermore, virulence factors seem to be reduced after LL-37 stress (depletion of histidine triad proteins) and a hypothetical decreased production rate of host-damaging H_2_O_2_ would explain the reduced synthesis of redox homeostasis proteins in pneumococci.

On the other hand, the regulation of pneumococcal adaptation in response to LL-37 seems to be mainly mediated by the transcriptional regulator GntR (SPD_1524) and the two-component systems, TCS05 CiaHR, TCS12 ComDE and TCS13 BlpHR (BlpS). In detail, GntR shows a substantial fold change at both timepoints, and corresponding RNA was also found to be considerably more abundant [45]. Next, both components of the CiaHR system, the sensor histidine kinase CiaH and the response regulator CiaR, are more abundant under stress on proteome and transcriptome level [45]. Finally, the response regulator part of ComDE (ComE) was increased on the protein level and the BlpHR two-component system was indirectly affected by an increase in BlpS protein and RNA in pneumococci after LL-37 stress. Indeed, this makes sense considering the complex interaction of Cia, Com and Blp regulatory systems [53], including the CiaHR-regulated degradation in ComC and BlpC signaling peptides by HtrA. After focusing on targets of the CiaHR regulon (probably activated by surface stress) [53] it becomes clear that it shows many of the effects known to be induced by its activity [43], among them, the accumulation of serine protease HtrA, foldase PrsA, the metabolic proteins MalP and MalQ and the unknown function protein SPD_0913.

Lastly, the data indicate that the unknown proteins SPD_0913, that was similarly induced on RNA level [45], and SPD_1515-17 (identified as less abundant uniquely on proteome level), play a potential role in innate immunity resistance. Perhaps SPD_1515-17 sensitizes pneumococci toward LL-37, and therefore a decreased abundance of this protein, and other proteins reduced in abundance after LL-37 stress, would result in a higher tolerance to the peptide. Backing up this hypothesis, SPD_1515-16 are annotated as membrane proteins, being potentially in contact with membrane-acting LL-37. However, the structures of these proteins are unknown, so that no statement can be made regarding a potential negative surface charge of this protein that would attract cationic AMPs.

For the two proteins DltD and LicD2, the genomic neighbor of significantly regulated LicD1, the role in LL-37 resistance was further supported by testing the sensitivity of D39 Δ*dltD* and Δ*licD2* deletion mutants towards the peptide. As expected from the literature [46], the Δ*dltD* strain was more susceptible to LL-37. Interestingly, the growth of the stressed Δ*licD2* mutant was even more reduced, indicating a role of the phosphorylcholine transferases LicD1-2 in LL-37 resistance, potentially by modifying the bacterial surface charge [24].

In conclusion, our study suggests that pneumococci may use adaptation mechanisms repulsion, export, and potentially degradation and interception to reduce the effects of the antimicrobial peptide LL-37. Certainly, this has yet to be confirmed in follow up experiments. However, as many of the presented candidates are changed at transcriptome and proteome levels in pneumococci, and several sequence similar proteins were already linked to AMP resistance in other bacteria, the presented candidates should be considered as highly relevant drug targets. 

Finally, global knowledge about bacterial resistance mechanisms to AMPs could be used to design new drugs, blocking the functional players of resistance. These components would sensitize pathogens to already existing AMPs in the human body. In fact, it was already shown that inhibition of the *dlt* operon, normally reducing the bacterial membrane charge by modifying teichoic acids with D-alanine, sensitizes Gram-positive *B. subtilis* to cell-wall acting-antibiotics [56]. Other targets for this approach could be the teichoic acid flippase TacF (SPD_1128), the phosphorylcholine transferases LicD1-2 or stress-induced efflux pumps that potentially export AMPs out of the bacteria. However, further research is needed to verify the role of AMP resistance candidates discussed here and design appropriate inhibitor molecules.

## Supplementary Materials

The following are available online at https://www.mdpi.com/2076-2607/8/3/413/s1.
