# Supplementary material for: Proteomic Adaptation of Streptococcus pneumoniae to the Human Antimicrobial Peptide LL-37
Source: Microorganisms. 2020 Mar 14;8(3):413. doi: 10.3390/microorganisms8030413 (PMC7143398; doi:10.3390/microorganisms8030413)
Supplement: Supplementary file 1 [file microorganisms-08-00413-s001.pdf]

Figure S1: Clustering of independent replicates

(A) Hierarchical clustering before filtering and imputation of missing values using standard settings of the Perseus software [1]

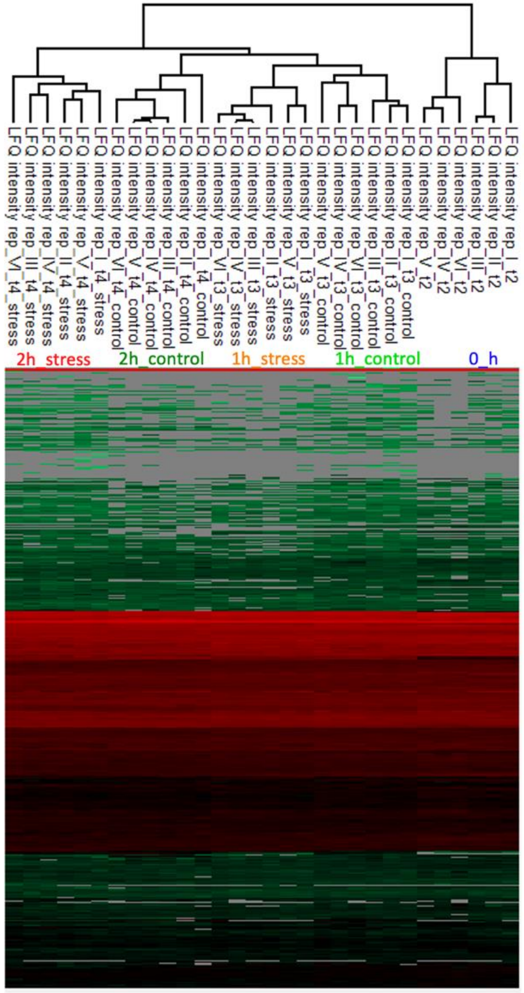

(B) Principal component analysis after filtering (5/6 in at least one exp. group) and imputation of missing values using standard settings of the Perseus software package [1]

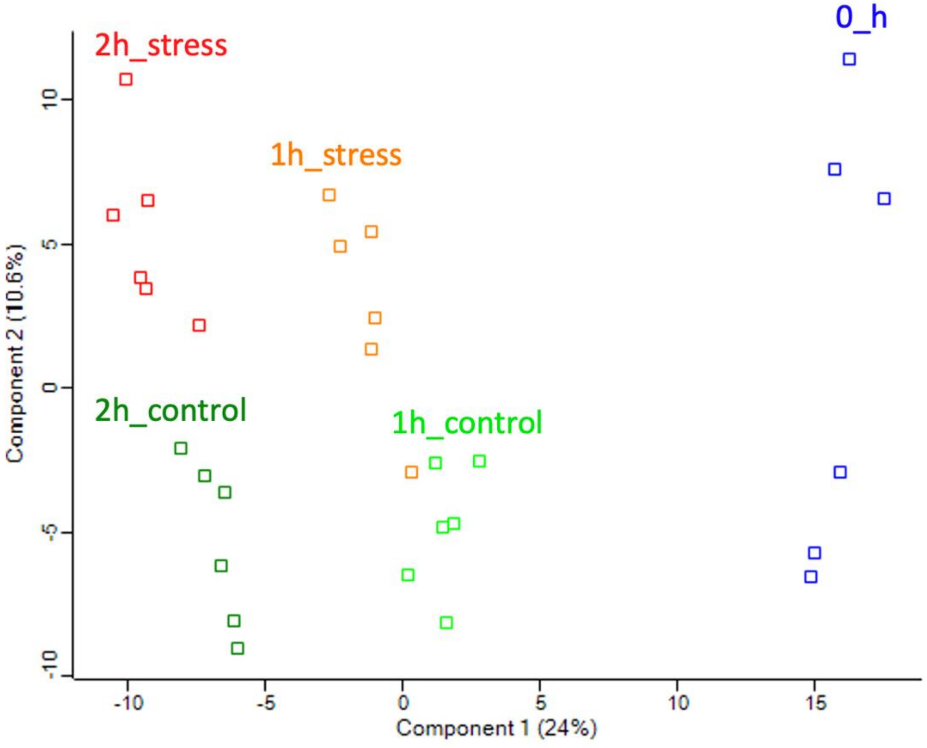

Figure S2: Multi scatter plot of 0 h samples

Pearson correlation

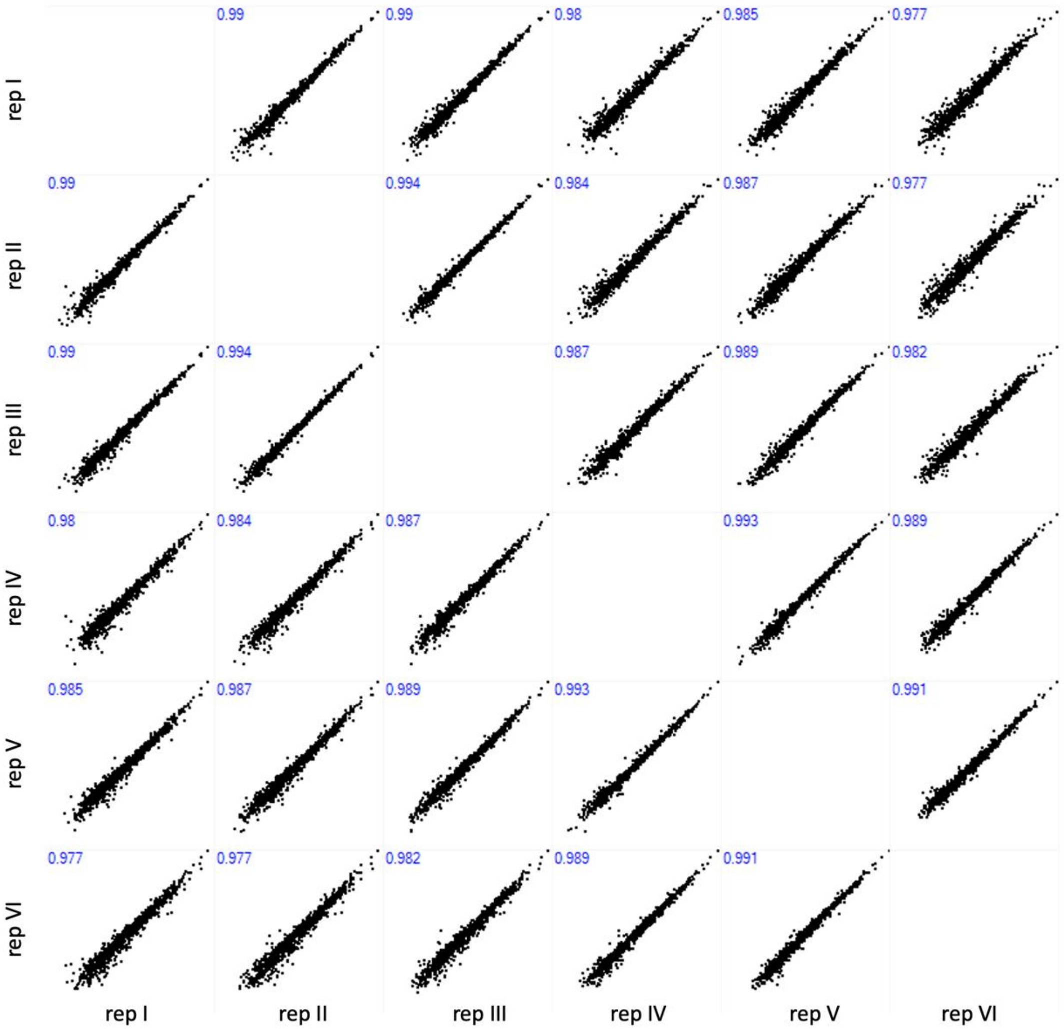

Figure S3: Multi scatter plot of 1 h control samples

Pearson correlation

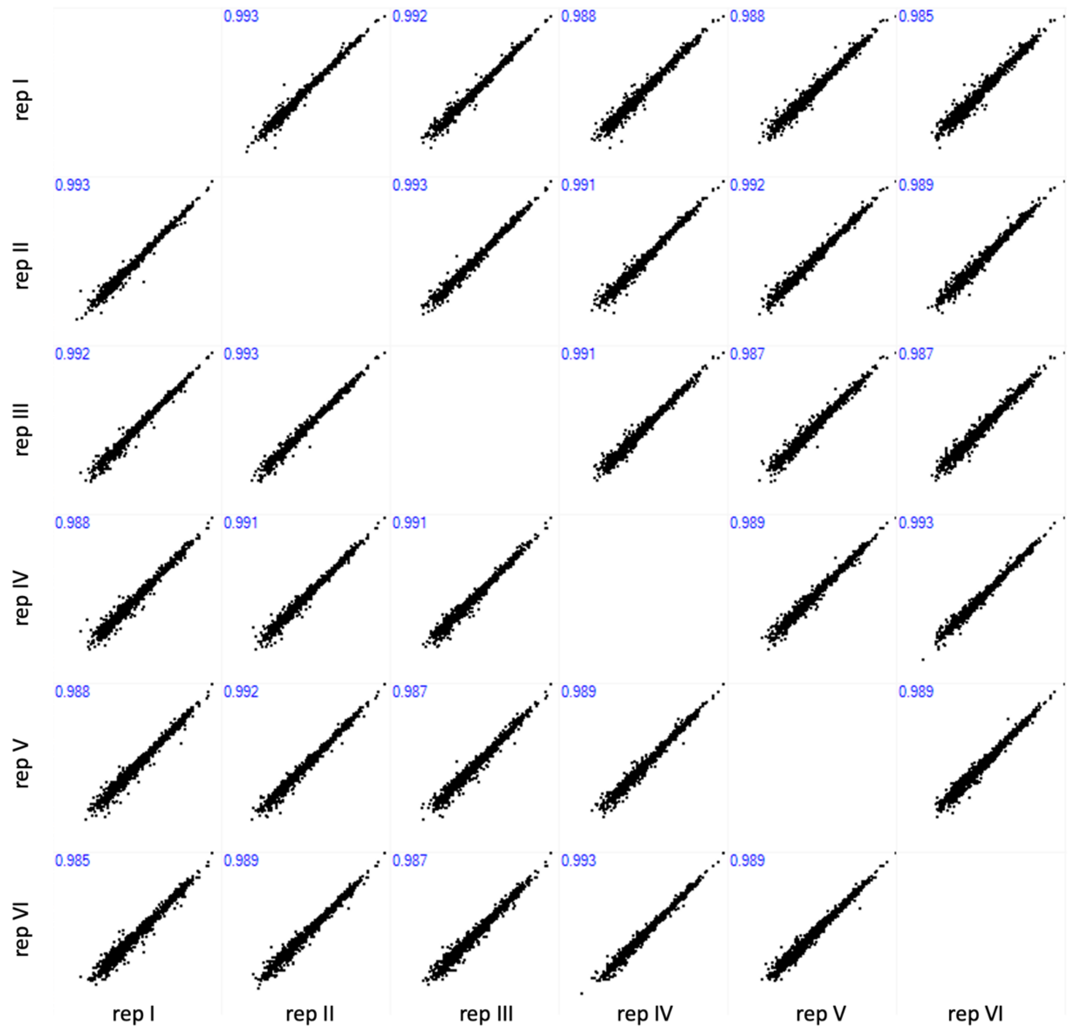

Figure S4: Multi scatter plot of 1 h stress samples

Pearson correlation

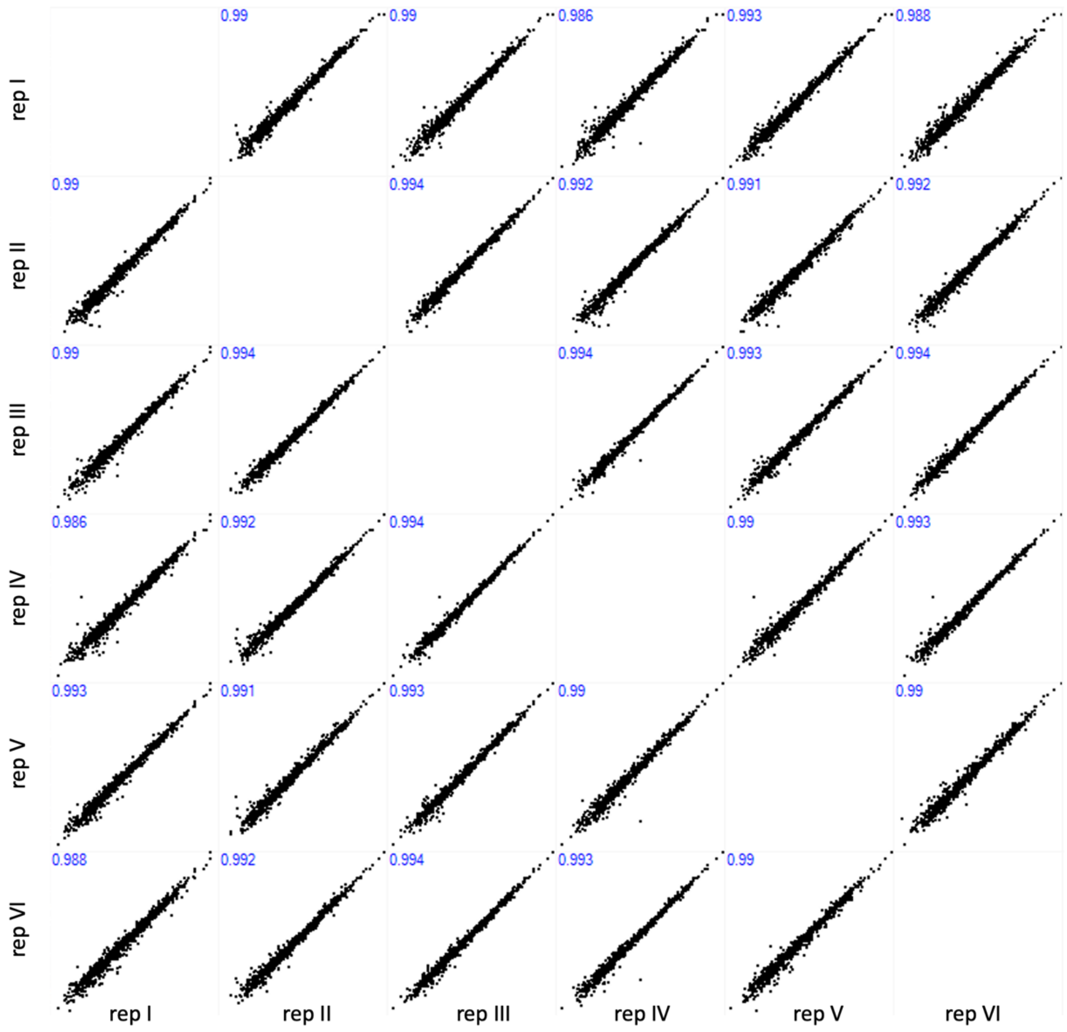

Figure S5: Multi scatter plot of 2 h control samples

Pearson correlation

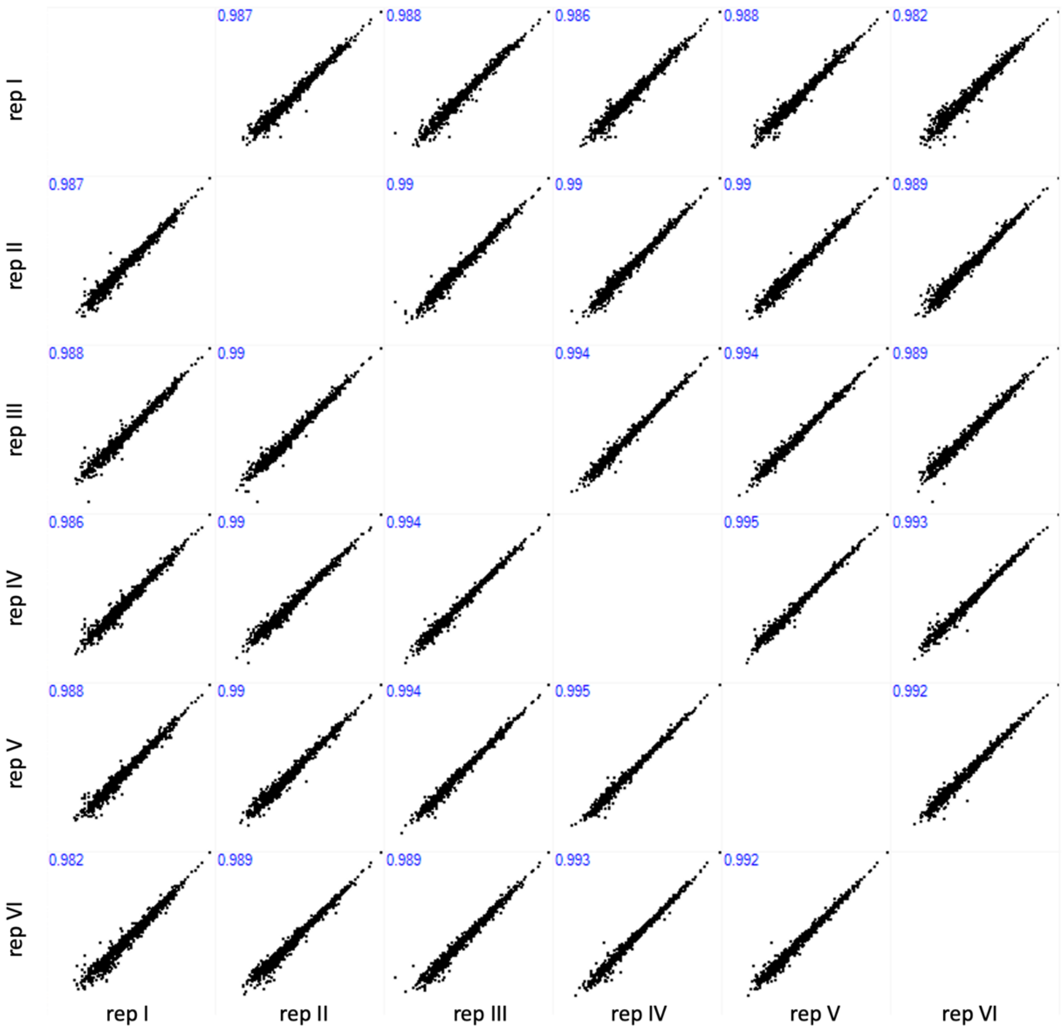

Figure S6: Multi scatter plot of 2 h stress samples

Pearson correlation

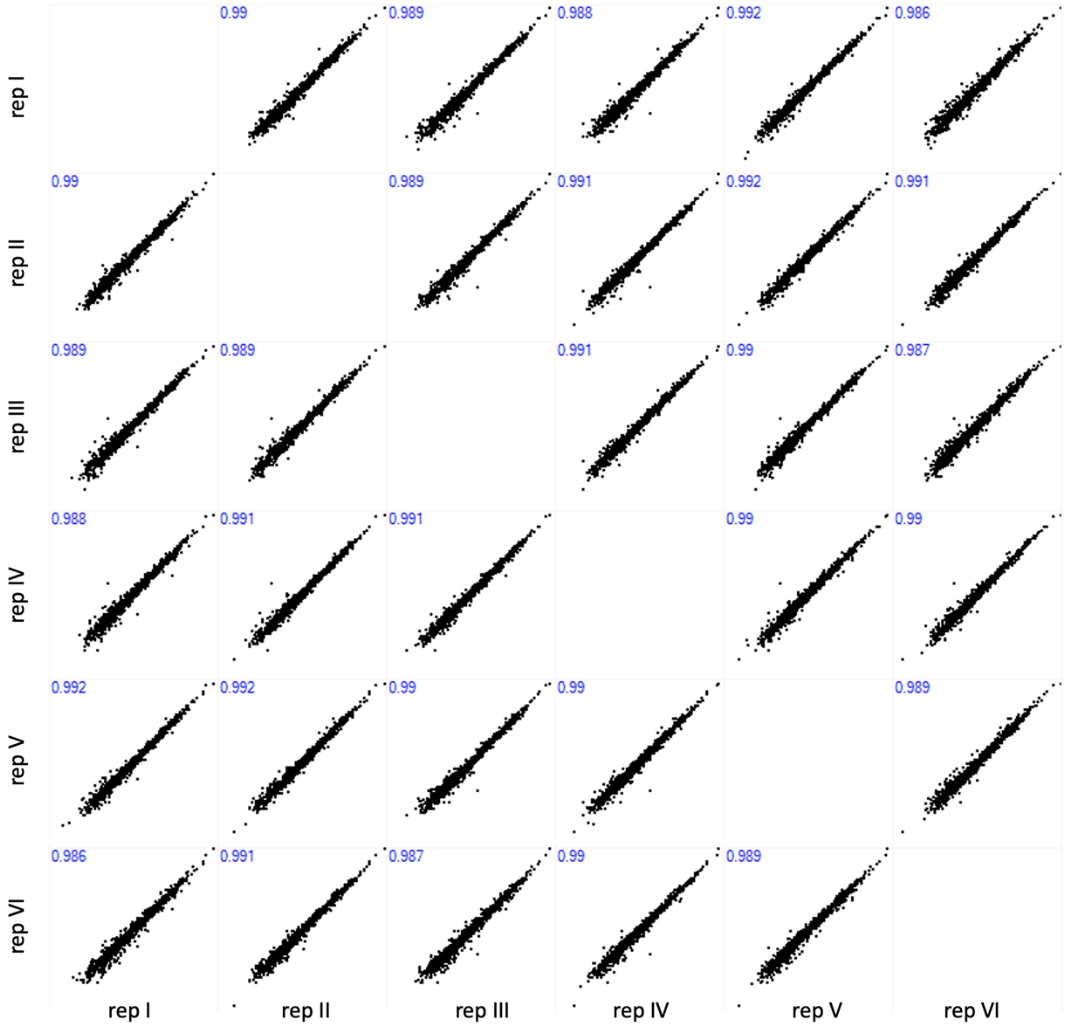

Table S1: Significant changes in protein abundance of *S. pneumoniae* D39 upon LL-37 exposure

locusID	protein	localization	protein fold change	
		PSORTdb_3.0 [2]	1 h stress	2 h stress
**14 transporters ↑**				
SPD_0076	Potassium uptake protein	Cytoplasmic Membrane	0.9	**2.9**
SPD_0107	Putative bacteriocin immunity protein	Cytoplasmic Membrane	0.9	**1.8**
SPD_0464	ABC transporter EcsA	Cytoplasmic Membrane	1.1	**1.6**
SPD_0684	Biotin transporter	Cytoplasmic Membrane	0.9	**4.3**
SPD_0686	Efflux transporter	Unknown	**1.7**	1.5
SPD_0687	ABC transporter	Cytoplasmic Membrane	1.4	**1.6**
SPD_0688	Efflux ABC transporter	Cytoplasmic Membrane	**1.8**	**2.1**
SPD_0861	Protein-export membrane protein SecG	Cytoplasmic Membrane	1.0	**2.2**
SPD_0887	Cationic amino acid transporter	Cytoplasmic Membrane	1.0	**5.4**
SPD_1169	Oligopeptide ABC transporter	Cytoplasmic Membrane	0.7	**2.8**
SPD_1214	ABC transporter	Cytoplasmic Membrane	1.9	**4.7**
SPD_1525	Multidrug ABC transporter	Cytoplasmic Membrane	**8.3**	**7.5**
SPD_1526	Putative ABC transporter	Cytoplasmic Membrane	on	**on**
SPD_2024	Thiamin ABC transporter	Cytoplasmic Membrane	0.5	**2.9**
**15 transporters ↓**				
SPD_0115	Putative efflux transporter	Cytoplasmic Membrane	**0.7**	**0.5**
SPD_0161	Putative divalent metal cation transporter	Cytoplasmic Membrane	**0.5**	**0.1**
SPD_0374	Putative ABC transporter	Cytoplasmic Membrane	-	**off**
SPD_0635	Cation-transporting ATPase	Cytoplasmic Membrane	**0.6**	**0.6**
SPD_0768	Putative AI-2E transporter	Cytoplasmic Membrane	**0.3**	2.6
SPD_1176	ABC transporter	Cytoplasmic Membrane	1.1	**0.6**

SPD_1263	ABC transporter	Cytoplasmic Membrane	0.2	**0.6**
SPD_1264	ABC transporter	Cytoplasmic Membrane	**0.3**	0.7
SPD_1465	Efflux ABC transporter	Cytoplasmic Membrane	**0.1**	0.7
SPD_1466	ABC transporter	Cytoplasmic Membrane	0.9	**0.7**
SPD_1514	ABC transporter	Cytoplasmic Membrane	**0.6**	**0.2**
SPD_1527	Putative sodium ABC exporter	Cytoplasmic Membrane	**0.4**	**0.3**
SPD_1528	Putative sodium ABC transporter	Cytoplasmic Membrane	**0.6**	0.7
SPD_1838	Preprotein translocase YajC subunit	Unknown	1.1	**0.6**
SPD_1997	Zinc ABC transporter AdcA	Cytoplasmic Membrane	**0.7**	**0.6**
**6 cell surface modification ↑**				
SPD_0099	Capsular polysaccharide biosynthesis protein CapD	Cytoplasmic Membrane	1.2	**1.5**
SPD_0873	Putative lyzozyme	Cytoplasmic	1.1	**1.6**
SPD_1076	Sortase SrtA	Unknown	**1.8**	**1.6**
SPD_1128	Putative teichoic acid flippase TacF	Cytoplasmic Membrane	0.8	**3.5**
SPD_1129	Phosphotransferase LicD1	Cytoplasmic	1.2	**1.8**
SPD_2002	DltD	Cytoplasmic Membrane	**1.6**	**1.7**
**1 cell surface modification ↓**				
SPD_0535	Serine/alanine-adding enzyme MurM	Cytoplasmic	**0.3**	1.1
**1 protease ↑**				
SPD_2068	serine protease HtrA	Unknown	**2.6**	**2.0**
**3 metabolism ↑**				
SPD_1932	Alpha-1,4 glucan phosphorylase MalP	Cytoplasmic Membrane	**1.8**	**2.2**
SPD_1933	4-alpha-glucanotransferase MalQ	Cytoplasmic	1.1	**2.3**

SPD_1664	Sugar PTS system	Cytoplasmic Membrane	1.0	**1.8**
**9 metabolism ↓**				
SPD_0404	Acetolactate synthase IlvB	Cytoplasmic	**0.3**	0.9
SPD_0736	Pyrimidine-nucleoside phosphorylase Pdp	Cytoplasmic	1.0	**0.6**
SPD_0895	Ferrochelatase HemH	Cytoplasmic	0.5	**0.3**
SPD_1078	L-lactate dehydrogenase L-LDH	Cytoplasmic	0.9	**0.7**
SPD_1089	Phosphopantothenoylcysteine decarboxylase CoaC	Unknown	**0.6**	1.1
SPD_1246	Glucosamine-6-phosphate deaminase NagB	Cytoplasmic	0.8	**0.6**
SPD_1532	Sugar PTS system	Cytoplasmic Membrane	**0.5**	1.5
SPD_1834	Aldehyde-alcohol dehydrogenase	Cytoplasmic	0.9	**0.7**
SPD_1934	Maltose ABC transporter MalX	Cytoplasmic Membrane	0.8	**0.6**
**7 virulence ↓**				
SPD_0579	Choline binding protein CbpL	Unknown	**0.5**	0.8
SPD_0854	Adherence and virulence protein A PavA	Cytoplasmic	**0.4**	1.3
SPD_0890	Pneumococcal histidine triad protein E PhtE	Unknown	**0.6**	**0.6**
SPD_1037	Histidine triad protein B	Unknown	**0.6**	0.8
SPD_1038	Pneumococcal histidine triad protein A PhpA	Unknown	0.7	**0.6**
SPD_1064	Putative hemolysin A	Cytoplasmic	**0.5**	0.7
SPD_1965	Choline binding protein PcpA	Extracellular	**0.5**	0.8
**12 other ↑**				
SPD_0170	Holliday junction ATP-dependent DNA helicase RuvA	Cytoplasmic	1.0	**1.5**
SPD_0476	Phosphotransferase enzyme family protein	Cytoplasmic	1.0	**1.6**
SPD_0534	Tributyrin esterase EstA	Unknown	1.1	**1.5**
SPD_0863	SsrA-binding protein (Small protein B) SmpB	Cytoplasmic	1.1	**1.5**

SPD_0868	Foldase PrsA	Cytoplasmic Membrane	**1.6**	**1.6**
SPD_0957	DNA primase DnaG	Cytoplasmic	1.0	**1.7**
SPD_1023	Tyrosine recombinase XerS	Cytoplasmic	1.1	**1.7**
SPD_1424	tRNA pseudouridine synthase A TruA	Cytoplasmic	1.0	**2.8**
SPD_1439	30S ribosomal protein S15 RpsO	Cytoplasmic	1.1	**1.5**
SPD_1546	Primosomal protein PriA	Cytoplasmic	1.5	**1.8**
SPD_1657	Tyrosine recombinase XerD-like	Cytoplasmic	1.0	**2.1**
SPD_1822	Ribosomal large subunit pseudouridine synthase	Cytoplasmic	1.3	**1.9**
**15 other ↓**				
SPD_0128	MutT/nudix family protein	Cytoplasmic	0.7	**0.5**
SPD_0886	Thioredoxin family protein Etrx2	Cellwall	0.7	**0.5**
SPD_0968	Acetyltransferase	Cytoplasmic Membrane	**0.5**	0.9
SPD_1041	Glutaredoxin-like protein NrdH	Unknown	**0.5**	0.4
SPD_1260	Methyltransferase	Cytoplasmic	1.1	**0.7**
SPD_1280	Cof family protein	Cytoplasmic	0.7	**0.7**
SPD_1301	NADPH-dependent FMN reductase	Cytoplasmic	0.3	**0.6**
SPD_1302	Putative oxidoreductase	Unknown	**0.6**	**0.4**
SPD_1375	Putative NADPH-dependent FMN reductase	Unknown	0.7	**0.6**
SPD_1464	Thioredoxin peroxidase Tpx PsaD	Unknown	1.0	**0.6**
SPD_1474	Cell division protein DivIVA	Cytoplasmic	**0.7**	0.8
SPD_1477	Cell division protein SepF	Cytoplasmic	1.1	**0.6**
SPD_1572	Ribosomal RNA small subunit methyltransferase E	Cytoplasmic Membrane	**0.6**	0.9
SPD_1590	Putative general stress protein 24	Cytoplasmic	0.7	**0.6**
SPD_2043	Secreted 45 kDa protein PcsB	Extracellular	0.7	**0.7**
**6 gene regulation ↑**				
SPD_0467	BlpS	Cytoplasmic	1.5	**1.8**

SPD_0701	Response regulator CiaR	Cytoplasmic	**1.6**	**1.6**
SPD_0702	Sensor histidine kinase CiaH	Cytoplasmic Membrane	**2.1**	2.1
SPD_1366	Putative general stress protein 13	Cytoplasmic	1.3	**2.2**
SPD_1524	Transcriptional regulator GntR	Unknown	**10.5**	**8.3**
SPD_2063	Response regulator ComE	Cytoplasmic	1.1	**1.5**
**5 gene regulation ↓**				
SPD_0309	Autoinducer-2 production protein LuxS	Cytoplasmic	0.7	**0.5**
SPD_0633	Putative transcriptional regulator CopY	Unknown	0.7	**0.5**
SPD_1448	Rrf2 family protein	Cytoplasmic	**0.2**	0.6
SPD_1535	Sucrose operon repressor ScrR	Cytoplasmic	**0.4**	1.2
SPD_1904	Arginine repressor ArgR	Cytoplasmic	**0.6**	**0.7**
**1 unknown ↑**				
SPD_0913	Unknown	Unknown	**2.6**	**2.8**
**10 unknown ↓**				
SPD_0039	Unknown	Cytoplasmic	0.9	**0.6**
SPD_0188	Unknown	Extracellular	**0.4**	1.0
SPD_0220	Unknown	Cytoplasmic Membrane	**0.3**	0.6
SPD_0714	Unknown	Cytoplasmic	0.8	**0.6**
SPD_0792	Putative lipoprotein	Unknown	0.7	**0.6**
SPD_1515	Putative membrane protein	Cytoplasmic Membrane	2.0	**0.4**
SPD_1516	Putative membrane protein	Cytoplasmic Membrane	0.7	**0.2**
SPD_1517	Unknown	Unknown	**0.5**	**0.3**
SPD_1588	Unknown	Cytoplasmic Membrane	**0.6**	0.7
SPD_1943	Unknown	Cytoplasmic Membrane	**0.1**	1.6

Table S2: Selected changes in protein abundance of *S. pneumoniae* D39 upon LL-37 exposure after comparison with transcriptomic and genomic data

Proteomic data (this study)					Gene regulation data [3,4]		Transcriptomic data [5]	
**locusID**	**protein**	**localization** PSORTdb_3.0 [2]	**protein fold change** 1 h stress	2h stress	**genomic regulator**	**locusID of regulator**	**RNA fold change** 15 min stress	30 min stress
**8 transporters ↑**								
SPD_0684	Biotin transporter	Cytoplasmic Membrane	0.9	**4.3**	RpoD	SPD_0958	**2.6**	**2.3**
SPD_0686	Efflux transporter	Unknown	**1.7**	1.5	GntR, RpoD	SPD_1524, SPD_0958	**4.1**	**5.5**
SPD_0687	ABC transporter	Cytoplasmic Membrane	1.4	**1.6**	GntR, RpoD	SPD_1524, SPD_0958	**5.1**	**5**
SPD_0688	Efflux ABC transporter	Cytoplasmic Membrane	**1.8**	**2.1**	GntR, RpoD	SPD_1524, SPD_0958	**4.6**	**6.1**
SPD_0887	Cationic amino acid transporter	Cytoplasmic Membrane	1.0	**5.4**	ArgR, RpoD	SPD_1904, SPD_0958		
SPD_1214	ABC transporter	Cytoplasmic Membrane	1.9	**4.7**	RpoD	SPD_0958	**1.5**	**1.6**
SPD_1525	Multidrug ABC transporter	Cytoplasmic Membrane	**8.3**	**7.5**	GntR, RpoD	SPD_1524, SPD_0958		
SPD_1526	Putative ABC transporter	Cytoplasmic Membrane	on	**on**	GntR, RpoD	SPD_1524, SPD_0958	**11.4**	**13**
**8 transporters ↓**								
SPD_0115	Putative efflux transporter	Cytoplasmic Membrane	**0.7**	**0.5**				
SPD_0161	Put. divalent metal cation transporter	Cytoplasmic Membrane	**0.5**	**0.1**	CodY	SPD_1412		
SPD_0374	Putative ABC transporter	Cytoplasmic Membrane	-	**off**	CcpA, ComE	SPD_1797, SPD_2063		
SPD_0635	Cation-transporting ATPase	Cytoplasmic Membrane	**0.6**	**0.6**	CopY, RpoD	SPD_0633, SPD_0958		
SPD_1514	ABC transporter	Cytoplasmic Membrane	**0.6**	**0.2**				
SPD_1527	Putative sodium ABC exporter	Cytoplasmic Membrane	**0.4**	**0.3**	CcpA, ComE, RpoD	SPD_1797, SPD_2063, SPD_0958	0.7	n. d.

SPD_1528	Putative sodium ABC transporter	Cytoplasmic Membrane	**0.6**	0.7	CcpA, ComE, RpoD	SPD_1797, SPD_2063, SPD_0958	**0.5**	**0.6**
SPD_1997	Zinc ABC transporter AdcA	Cytoplasmic Membrane	**0.7**	**0.6**	AdcR, RpoD	SPD_2000, SPD_0958		
**4 cell surface modification ↑**								
SPD_1076	Sortase SrtA	Unknown	**1.8**	**1.6**				
SPD_1128	Putative teichoic acid flippase TacF	Cytoplasmic Membrane	0.8	**3.5**				
SPD_1129	Phosphotransferase LicD1	Cytoplasmic	1.2	**1.8**				
SPD_2002	DltD	Cytoplasmic Membrane	**1.6**	**1.7**			**1.6**	n. d.
**1 protease ↑**								
SPD_2068	serine protease HtrA	Unknown	**2.6**	**2.0**	CiaR	SPD_0701	**3**	**2.3**
**2 metabolism ↑**								
SPD_1932	Alpha-1,4 glucan phosphorylase MalP	Cytoplasmic Membrane	**1.8**	**2.2**	CcpA, MalR, RpoD, CiaR	SPD_1797, SPD_1938, SPD_0958, SPD_0701	**3.2**	**2.6**
SPD_1933	4-alpha-glucanotransferase MalQ	Cytoplasmic	1.1	**2.3**	CcpA, MalR, RpoD, CiaR	SPD_1797, SPD_1938, SPD_0958, SPD_0701	**3.4**	**2.9**
**4 metabolism ↓**								
SPD_0404	Acetolactate synthase IlvB	Cytoplasmic	**0.3**	0.9	CodY, RpoD	SPD_1412, SPD_0958	0.7	**0.6**
SPD_1532	Sugar PTS system	Cytoplasmic Membrane	**0.5**	1.5	CcpA, RpoD, ScrR	SPD_1797, SPD_0958, SPD_1535		
SPD_1834	Aldehyde-alcohol dehydrogenase	Cytoplasmic	0.9	**0.7**	CcpA, Rex, RpoD	SPD_1797, SPD_0976, SPD_0958	**0.6**	**0.4**
SPD_1934	ABC transporter MalX	Cytoplasmic Membrane	0.8	**0.6**	CcpA, MalR, RpoD	SPD_1797, SPD_1938, SPD_0958	**0.6**	n. d.

**3 virulence ↓**								
SPD_0890	Pneumococcal histidine triad protein E PhtE	Unknown	**0.6**	**0.6**	AdcR	SPD_2000	n. d.	**0.6**
SPD_1037	Histidine triad protein B	Unknown	**0.6**	0.8	AdcR, RpoD	SPD_2000, SPD_0958		
SPD_1038	Pneumococcal histidine triad protein A PhpA	Unknown	0.7	**0.6**	AdcR, RpoD	SPD_2000, SPD_0958		
**2 others ↑**								
SPD_0863	SsrA-binding protein (Small protein B) SmpB	Cytoplasmic	1.1	**1.5**			**1.5**	**1.7**
SPD_0868	Foldase PrsA	Cytoplasmic Membrane	**1.6**	**1.6**	CiaR	SPD_0701	1.3	n. d.
**6 others ↓**								
SPD_1301	NADPH-dependent FMN reductase	Cytoplasmic	0.3	**0.6**	RpoD	SPD_0958		
SPD_1302	Putative oxidoreductase	Unknown	**0.6**	**0.4**	RpoD	SPD_0958		
SPD_1464	Thioredoxin peroxidase Tpx PsaD	Unknown	1.0	**0.6**			n. d.	**0.5**
SPD_1474	Cell division protein DivIVA	Cytoplasmic	**0.7**	0.8				
SPD_1477	Cell division protein SepF	Cytoplasmic	1.1	**0.6**				
SPD_1590	Putative general stress protein 24	Cytoplasmic	0.7	**0.6**	RpoD	SPD_0958		
**5 gene regulation ↑**								
SPD_0467	BlpS	Cytoplasmic	1.5	**1.8**			**1.6**	**1.8**
SPD_0701	Response regulator CiaR	Cytoplasmic	**1.6**	**1.6**	CiaR	SPD_0701	**2.5**	**2.1**
SPD_0702	Sensor histidine kinase CiaH	Cytoplasmic Membrane	**2.1**	2.1	CiaR	SPD_0701	**2.4**	n. d.
SPD_1524	Transcriptional regulator GntR	Unknown	**10.5**	**8.3**	GntR, RpoD	SPD_1524, SPD_0958	**7.2**	**9.1**
SPD_2063	Response regulator ComE	Cytoplasmic	1.1	**1.5**	ComE, RpoD	SPD_2063, SPD_0958	n. d.	0.7

**3 gene regulation ↓**								
SPD_0633	Putative transcriptional regulator CopY	Unknown	0.7	**0.5**	CopY, RpoD	SPD_0633, SPD_0958		
SPD_1535	Sucrose operon repressor ScrR	Cytoplasmic	**0.4**	1.2				
SPD_1904	Arginine repressor ArgR	Cytoplasmic	**0.6**	**0.7**	ArgR, RpoD	SPD_1904, SPD_0958		
**1 unknown ↑**								
SPD_0913	Unknown	Unknown	**2.6**	**2.8**	CiaR	SPD_0701	**3.3**	**2.7**
**5 unknown ↓**								
SPD_0220	Unknown	Cytoplasmic Membrane	**0.3**	0.6	CcpA	SPD_1797	**0.6**	n. d.
SPD_1515	Putative membrane protein	Cytoplasmic Membrane	2.0	**0.4**				
SPD_1516	Putative membrane protein	Cytoplasmic Membrane	0.7	**0.2**				
SPD_1517	Unknown	Unknown	**0.5**	**0.3**				
SPD_1588	Unknown	Cytoplasmic Membrane	**0.6**	0.7	RpoD	SPD_0958		

References

1. Tyanova, S.; Temu, T.; Sinitcyn, P.; Carlson, A.; Hein, M.Y.; Geiger, T.; Mann, M.; Cox, J. The Perseus computational platform for comprehensive analysis of (prote)omics data. *Nature Methods* **2016**, *13*, 731-740, doi:10.1038/nMeth.3901.

2. Peabody, M.A.; Laird, M.R.; Vlasschaert, C.; Lo, R.; Brinkman, F.S. PSORTdb: expanding the bacteria and archaea protein subcellular localization database to better reflect diversity in cell envelope structures. *Nucleic Acids Res.* **2016**, *44*, D663-D668, doi:10.1093/nar/gkv1271.

3. Slager, J.; Aprianto, R.; Veening, J.W. Deep genome annotation of the opportunistic human pathogen *Streptococcus pneumoniae* D39. *Nucleic Acids Res.* **2018**, *46*, 9971-9989, doi:10.1093/nar/gky725.

4. Halfmann, A.; Kovacs, M.; Hakenbeck, R.; Bruckner, R. Identification of the genes directly controlled by the response regulator CiaR in *Streptococcus pneumoniae*: five out of 15 promoters drive expression of small non-coding RNAs. *Mol. Microbiol.* **2007**, *66*, 110-126, doi:10.1111/j.1365-2958.2007.05900.x.

5. Majchrzykiewicz, J.A.; Kuipers, O.P.; Bijlsma, J.J. Generic and specific adaptive responses of *Streptococcus pneumoniae* to challenge with three distinct antimicrobial peptides, bacitracin, LL-37, and nisin. *Antimicrob. Agents Chemother.* **2010**, *54*, 440-451, doi:10.1128/AAC.00769-09.
